# Development and characterization of efficient xylose utilization strains of *Zymomonas mobilis*

**DOI:** 10.1186/s13068-021-02082-x

**Published:** 2021-12-04

**Authors:** Jiyun Lou, Jingwen Wang, Yongfu Yang, Qing Yang, Runxia LI, Mimi Hu, Qiaoning He, Jun Du, Xia Wang, Mian Li, Shihui Yang

**Affiliations:** 1 State Key Laboratory of Biocatalysis and Enzyme Engineering, Environmental Microbial Technology Center of Hubei Province, and School of Life Sciences, Hubei University, Wuhan, 430062 China; 2 China Biotech Fermentation Industry Association, Beijing, 100833 China; 3 Zhejiang Huakang Pharmaceutical Co., Ltd., Kaihua County, Zhejiang, China

**Keywords:** *Zymomonas mobilis*, Xylose utilization, Xylose isomerase pathway, Adaptive laboratory evolution (ALE), Genome resequencing, RNA-Seq

## Abstract

**Background:**

Efficient use of glucose and xylose is a key for the economic production of lignocellulosic biofuels and biochemicals, and different recombinant strains have been constructed for xylose utilization including those using *Zymomonas mobilis* as the host. However, the xylose utilization efficiency still needs to be improved. In this work, the strategy of combining metabolic engineering and adaptive laboratory evolution (ALE) was employed to develop recombinant *Z. mobilis* strains that can utilize xylose efficiently at high concentrations, and NGS-based genome resequencing and RNA-Seq transcriptomics were performed for strains evolved after serial transfers in different media to understand the impact of xylose and differences among strains with different xylose-utilization capabilities at molecular level.

**Results:**

Heterologous genes encoding xylose isomerase and xylulokinase were evaluated, which were then introduced into xylose-utilizing strain *Z. mobilis* 8b to enhance its capacity of xylose utilization. The results demonstrated that the effect of three xylose isomerases on xylose utilization was different, and the increase of copy number of xylose metabolism genes can improve xylose utilization. Among various recombinant strains constructed, the xylose utilization capacity of the recombinant strain 8b-*RsXI-xylB* was the best, which was further improved through continuous adaption with 38 transfers over 100 days in 50 g/L xylose media. The fermentation performances of the parental strain 8b, the evolved 8b-S38 strain with the best xylose utilization capability, and the intermediate strain 8b-S8 in different media were compared, and the results showed that only 8b-S38 could completely consume xylose at 50 g/L and 100 g/L concentrations. In addition, the xylose consumption rate of 8b-S38 was faster than that of 8b at different xylose concentrations from 50 to 150 g/L, and the ethanol yield increased by 16 ~ 40%, respectively. The results of the mixed-sugar fermentation also demonstrated that 8b-S38 had a higher xylose consumption rate than 8b, and its maximum ethanol productivity was 1.2 ~ 1.4 times higher than that of 8b and 8b-S8. Whole-genome resequencing identified three common genetic changes in 8b-S38 compared with 8b and 8b-S8. RNA-Seq study demonstrated that the expression levels of genes encoding chaperone proteins, ATP-dependent proteases, phage shock proteins, ribosomal proteins, flagellar operons, and transcriptional regulators were significantly increased in xylose media in 8b-S38. The up-regulated expression of these genes may therefore contribute to the efficient xylose utilization of 8b-S38 by maintaining the normal cell metabolism and growth, repairing cellular damages, and rebalancing cellular energy to help cells resist the stressful environment.

**Conclusions:**

This study provides gene candidates to improve xylose utilization, and the result of expressing an extra copy of xylose isomerase and xylulokinase improved xylose utilization also provides a direction for efficient xylose-utilization strain development in other microorganisms. In addition, this study demonstrated the necessity to combine metabolic engineering and ALE for industrial strain development. The recombinant strain 8b-S38 can efficiently metabolize xylose for ethanol fermentation at high xylose concentrations as well as in mixed sugars of glucose and xylose, which could be further developed as the microbial biocatalyst for the production of lignocellulosic biofuels and biochemicals.

**Supplementary Information:**

The online version contains supplementary material available at 10.1186/s13068-021-02082-x.

## Background

Second-generation bioethanol using lignocellulosic biomass is a promising sustainable alternative to fossil fuels to reduce greenhouse gas [1]. It does not have any direct competition with food production like first-generation bioethanol usually produced from sugar and amylaceous plants, which has potential ethical issues [2]. Lignocellulosic biomass, such as corn stover, wheat straw, rice straw, and forest residues, is the most abundant renewable resource in the world, which is considered as a sustainable feedstock for biofuel production. Lignocellulosic biomass primarily contains cellulose, hemicellulose, and lignin [3]. Cellulose and hemicellulose are polysaccharides that can be converted into fermentable hexoses and pentoses such as glucose and xylose, which are the most abundant monosaccharides accounting for 60 ~ 70% and 30 ~ 40% of lignocellulosic hydrolysates, respectively [3–5]. However, the high production cost associated with lignocellulose deconstruction still hampered its economic production, and the efficient utilization of both hexoses and pentoses released from lignocellulosic biomass for large-scale fermentation is essential for industrial application [1, 5].

Native ethanologens, such as *Saccharomyces cerevisiae* and *Zymomonas mobilis*, can produce bioethanol [5, 6]. Compared with *S. cerevisiae*, the Gram-negative facultative anaerobic ethanologenic bacterium *Z. mobilis* possesses a unique Entner–Doudoroff (ED) pathway that converts 97% glucose to ethanol [7, 8]. Many excellent characteristics, such as high specific glucose uptake rate, high ethanol titer, high ethanol tolerance up to 16% (v/v), and low aeration cost make it an ideal candidate for industrial applications [9–11].

However, wild-type *Z. mobilis* can use only a few sugars like glucose and fructose as the carbon source, but cannot utilize pentoses such as xylose, which is the second most abundant sugar in pretreated lignocellulosic hydrolysates [11, 12]. Xylose assimilation and metabolism pathway have been introduced into *Z. mobilis*, enabling the strain to utilize xylose as fermentable substrates to produce ethanol [7, 13–16]. Xylose assimilation genes (xylose isomerase *xylA*, xylulokinase *xylB*) and pentose metabolism genes (transketolase *tktA*, transaldolase *talB*) from *Escherichia coli* were first expressed in wild-type *Z. mobilis* CP4 [7]. *Z. mobilis* 8b is a robust xylose utilization recombinant by expressing heterologous *xylAB* and *talB-tktA* genes for xylose utilization as well as truncating the endogenous lactate dehydrogenase gene (*ldh*) to promote ethanol titer [13]. A few studies have then been carried out in *Z. mobilis* 8b to further improve xylose utilization [17, 18], and the complete genome sequence and the expression pattern of plasmids of strain 8b have been analyzed [19]. However, problems remain to be resolved, such as the low xylose uptake rate, prolonged fermentation time, and incomplete xylose utilization in lignocellulosic hydrolysates especially those containing inhibitory compounds [12, 14–16].

Metabolic engineering has been playing increasingly important roles in strain development by altering the metabolic pathways [20, 21], and recombinant microorganisms have been constructed for lignocellulosic biofuel production [22, 23] including those with extended substrate utilization spectra such as xylose, arabinose, and galactose [24–26]. Besides the metabolic engineering strategy, adaptive laboratory evolution (ALE) is another powerful approach to generate random mutations in multiple genes in a controlled laboratory setting [27]. By combining rational metabolic rewiring with the laboratory evolution, heterotrophic organism *E. coli* was enabled to produce its biomass carbon from CO_2_ directly [28, 29]. It has been utilized in *Z. mobilis* as well to obtain strains with efficient xylose utilization [15–18, 30]. For example, an engineered strain of *Z. mobilis* showed markedly improved xylose utilization ability and lower xylitol production than its parental strain after adaptation over 80 days through 30 serial transfers [16]. ALE was further combined with high-throughput sequencing to characterize the potential genetic changes responsible for the pentose utilization of an adapted *Z. mobilis* strain [15]. Robust strain *Z. mobilis* 8b was evolved through ALE as well but in the presence of acetate and 2-deoxyglucose, or pretreated corn stover to generate strains with efficient xylose utilization in the presence of glucose and other model inhibitory compounds [17, 18]. Although these studies demonstrated the potential of developing *Z. mobilis* strains for efficient xylose metabolism through metabolic engineering and ALE, mutant strains could not simultaneously utilize glucose and xylose efficiently.

Since xylose reductase and xylitol dehydrogenase are usually cofactor dependent, overexpression of these genes will result in the imbalance of cofactors in *Z. mobilis* and lead to the accumulation of the deleterious intermediate xylitol [31]. Therefore, in the present study, we evaluated the effect of overexpression of xylose isomerase (XI) and xylulokinase in xylose-utilization recombinant strain *Z. mobilis* 8b to avoid this problem, which can convert xylose to xylulose-5-phosphate without cofactors. Considering that the existing XI activity, encoded by *xylA* in *Z. mobilis* 8b is significantly lower than other activities of xylose-metabolism enzymes [16], *PiXI* gene from *Piromyces* sp. E2 [32], *RsXI* from the protists in the *Reticulitermes speratus* hindgut [33], and *RuXI* from bovine rumen contents [34], which were previously proved to function in microorganisms with higher enzyme activities, were selected for constructing in *Z. mobilis*. Then, ALE was further applied to obtain efficient xylose utilization strains. Cell growth, sugar consumption and ethanol production of the evolved strains in pure glucose, xylose of different concentrations and mixed sugars of glucose and xylose were analyzed and compared with the parental strain *Z. mobilis* 8b. Finally, the molecular mechanism and genetic targets contributing to the improved xylose utilization were investigated using next-generation sequencing (NGS)-based genome resequencing and RNA-Seq transcriptomics.

## Results and discussion

### Evaluation of different xylose isomerase and xylulokinase in *Z. mobilis* ZM4

To verify the ability of different xylose isomerases in improving the xylose utilization of *Z. mobilis*, three different xylose isomerases were chosen to express in *Z. mobilis* ZM4. Since *Z. mobilis* ZM4 lacks a complete pentose phosphate pathway (PPP), which is necessary for xylose utilization, the plasmid pZM41 from *Z. mobilis* 8b containing two enzymes (TalB and TktA) of PPP [19] were extracted and electroporated into *Z. mobilis* ZM4 to obtain a new strain named *Z. mobilis* ZMP with the complete PPP. Then, genes encoding xylose isomerase (XI) and xylulokinase (XK) were cloned into the shuttle vector pEZ15Asp under the control of the tetracycline-inducible promoter P*tet*, which were then electroporated into *Z. mobilis* ZMP strain to generate the potential xylose-utilization recombinant strains (Additional file 1: Fig. S1).

**Fig. 1:**
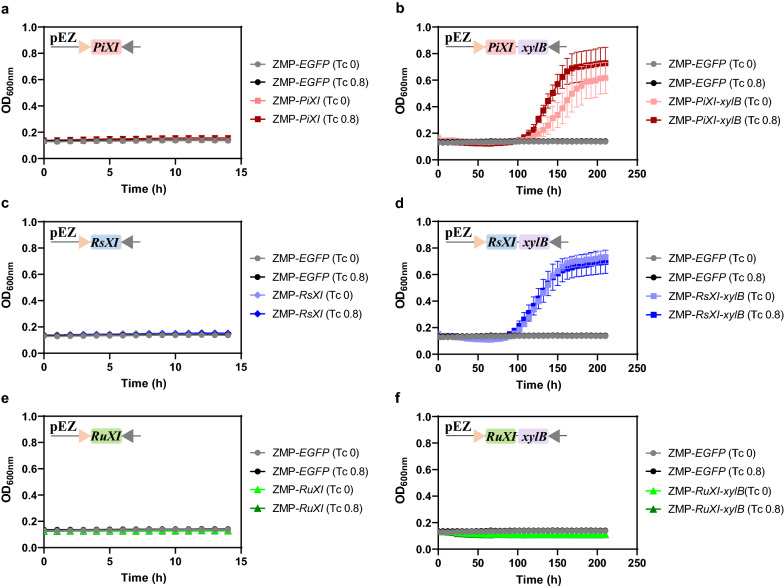
Cell growth of strain ZMP-*PiXI* (**a**), ZMP-*PiXI*-*xylB* (**b**), ZMP-*RsXI* (**c**), ZMP-*RsXI*-*xylB* (**d**), ZMP-*RuXI* (**e**) and ZMP-*RuXI*-*xylB* (**f**) in RMX5. Strains were cultured in 300 μL RMX5 with 50 μg/mL spectinomycin in 500 μL microporous honeycomb panel by Bioscreen C instrument at 30 °C. The induced concentration of tetracycline is 0 and 0.8 μg/mL, the plate was shaken before measuring the OD_600 nm_ every 3 min. Three replicates were performed for each experiment. The diagram in each panel represented plasmid constructs expressing XI alone or both XI and XK genes controlled by P*tet* promoter (pink triangle) and *rrnB* T1 terminator (grey triangle). *PiXI*, *RsXI*, and *RuXI* represented genes encoding xylose isomerase (XI) from *Piromyces* sp. E2, protists in the *Reticulitermes speratus* hindgut, and bovine rumen contents, respectively; *xylB* was xylulokinase gene from *E. coli*. Plasmid containing the reporter gene *EGFP* was used as the control

Fermentation was performed to evaluate the effect of different xylose utilization genes in ZMP. The results of the growth on 50 g/L xylose indicated that with only one XI gene (*PiXI*, *RsXI*, or *RuXI*) in *Z. mobilis* ZMP, the strains could not utilize xylose (Fig. 1a, c, e). When XK gene was further incorporated and co-expressed with XI gene, recombinant stains ZMP-*PiXI-xylB* and ZMP-*RsXI-xylB* exhibited great xylose utilization potential, even without tetracycline induction (Fig. 1b, d, f). When the tetracycline concentration increased to 0.8 μg/mL, both mutants can grow to a final OD_600_ value around 0.70 (Fig. 1b, d). However, strain ZMP-*RuXI*-*xylB* hardly grew with xylose as the sole carbon source, even with 0.8 μg/mL tetracycline induction (Fig. 1f).

Although xylose metabolic pathways have been introduced into *Z. mobilis* ZMP, the low efficiency of RuXI may limit the xylose fermentation. As reported in previous results in *S. cerevisiae*, the optimum pH and temperature of RuXI from bovine rumen were 7.0 and 60 °C, respectively [34]. Hence, its activity in *Z. mobilis* cultured at a temperature of 30 °C that is lower than its optimum temperature may be affected. In addition, the potential for inappropriate folding of the bovine rumen enzymes by the prokaryotic host may also result in a reduction in efficiency. However, PiXI from eukaryotic *Piromyces* sp*.* E2 [32] and RsXI from the protists in the termite hindgut [33] combined with *xylB* gene encoding xylulokinase were proved to be more effective in xylose utilization in ZMP (Fig. 1). Thus, *PiXI-xylB* and *RsXI-xylB* were selected for further study.

### Construction of efficient xylose-utilization recombinant strains using *Z. mobilis* 8b

**Fig. 2:**
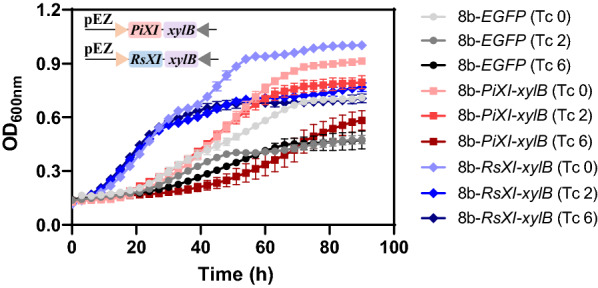
Cell growth of recombinant strains of 8b-*PiXI*-*xylB* and 8b-*RsXI*-*xylB* in RMX5. Strains were cultured in 300 μL RMX5 with 50 μg/mL spectinomycin in 500 μL microporous honeycomb panel by Bioscreen C instrument at 30 °C. The induced concentration of tetracycline is 0, 2, and 6 μg/mL, and the plate was shaken before measuring the OD_600 nm_ every 3 min. Three replicates were performed for each experiment. The diagram in the panel represented plasmid constructs expressing both XI and XK genes controlled by the P*tet* promoter (pink triangle) and *rrnB* T1 terminator (grey triangle). *PiXI* and *RsXI* represented the xylose isomerase (XI) genes from *Piromyces* sp. E2, and protists in the *Reticulitermes speratus* hindgut, respectively; *xylB* was xylulokinase gene from *E. coli*. Plasmid containing the reporter gene *EGFP* was used as the control

*Z. mobilis* 8b has been engineered by integration of *E. coli talB-tktA* and *xylA-xylB* genes for xylose utilization [7, 13]. Considering the fact that XI pathway bypasses the cofactor imbalance and is beneficial for xylose utilization, XI gene *PiXI* or *RsXI* with *xylB* were electroporated into 8b to explore their effect on xylose utilization capacity. As demonstrated in Fig. 2, the engineered strain 8b-*RsXI-xylB* and 8b-*PiXI-xylB* demonstrated better xylose utilization. The highest biomass in terms of OD_600_ of 1.00 was obtained for strain 8b-*RsXI-xylB*, which is 1.40 times and 1.10 times than those of the control strain 8b-*EGFP* (OD_600_ of 0.71) and another recombinant strain 8b-*PiXI-xylB* (OD_600_ of 0.91) without tetracycline induction, respectively. With the tetracycline concentration increasing from 0 to 6 μg/mL, the biomass decreased in all three strains. However, under 6 μg/mL of tetracycline, the growth in engineered 8b-*RsXI*-*xylB* strain still had about 1.50 times biomass of the control strain (Fig. 2).

Although several studies have reported that the increase of copy number of xylose isomerase and xylulokinase genes cannot promote the xylose utilization ability directly [35], the functional expression of *PiXI* and *xylB* in *Z. mobilis* 8b did promote the biomass of engineered strain grown in the medium with xylose as the sole carbon source. XI catalyzes the cofactor-independent reaction that directly converts xylose to xylulose and thus can avoid xylitol production [36]. Different resources of XI have different efficiency in xylose utilization in *Z. mobilis*. The results indicated that RsXI may be more efficient for *Z. mobilis* 8b. Therefore, strain 8b-*RsXI-xylB* was used for further adaptive laboratory evolution. Moreover, the results above showed that with the concentration of tetracycline inducer increased, the growth of the strain was inhibited although the engineered xylose-utilization strain 8b contains a tetracycline resistance gene [13], thus no tetracycline was added in subsequent experiments.

### Adaptive laboratory evolution of *Z. mobilis* 8b-*RsXI*-*xylB*

**Fig. 3:**
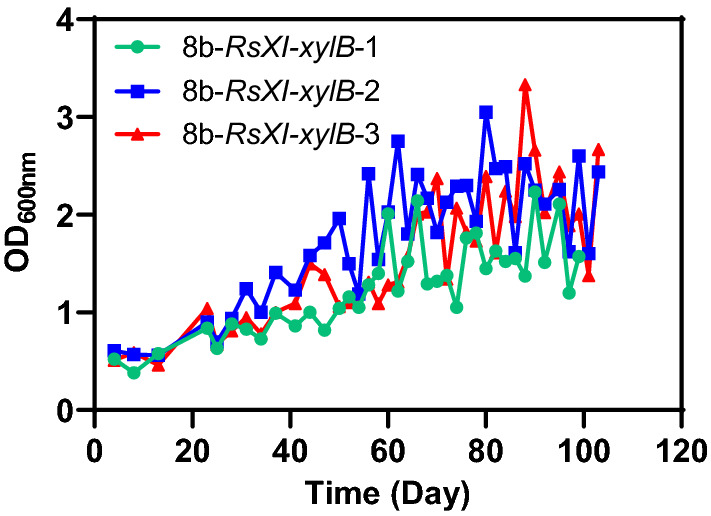
Adaptive laboratory evolution of 8b-*RsXI*-*xylB* with 50 g/L xylose as the selection pressure. Each point represents one round of transfer. 38 transfers were conducted within about 100 days

Metabolic engineering combined with ALE has proven to be a successful approach for strains development with ideal targets through natural selection of beneficial genetic variations [16–18, 37, 38]. In this work, ALE was implemented as well to achieve the highly efficient xylose utilization capability in *Z. mobilis*. The engineered strain 8b-*RsXI-xylB* was sub-cultivated in triplicates and subjected to 50 g/L xylose as the selection pressure. The total evolution lasted for about 100 days with at least 38 transfers (Fig. 3).

During the first 30 days, the growth of three replicates was very poor with the maximum OD_600_ value lower than 1.0. After that, the biomass in terms of OD_600_ value in these three replicates was significantly increased in the next 30 days. With the extended adaption process in the following days, all three replicates still had a slow increase of OD_600_. Notably, replicate of strain 8b-*RsXI-xylB*-2 demonstrated a steady improvement in xylose-utilization capability during the ALE process (Fig. 3). The biomass of 8b-*RsXI-xylB*-2 in terms of OD_600_ increased to 2.30 after 60 days of adaption, which was about 3 times higher than the initial OD_600_ of 0.55 and higher than those of other two replicates at the same time. After 80 days of adaption, strain 8b-*RsXI-xylB*-2 reached an OD_600_ value of 2.70, about 5 times than the initial inoculum OD_600_.

**Fig. 4:**
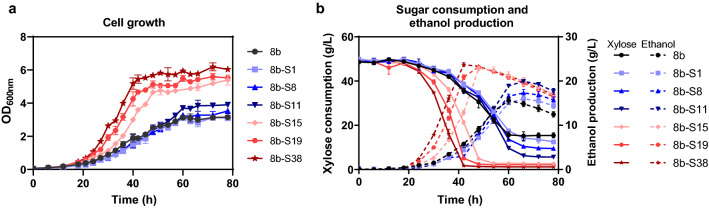
Cell growth, sugar consumption, and ethanol production of mutant strains selected at different time points during ALE in RMX5. 8b-S1, 8b-S8, 8b-S11, 8b-S19, and 8b-S38 were selected after the 1st, 8th, 11th, 15th, 19th, and 38th transfers, respectively. Strains were cultured in 40 mL RMX5 in 50-mL shake flasks at 100 rpm and 30 °C. Spectinomycin at a final concentration of 50 μg/mL was added for recombinant strains. Three replicates were performed for each experiment

Fermentation of strain 8b-*RsXI-xylB*-2 in different stages (1st,11th, 15th, 19th, and 38th transfers) from ALE were carried out under 50 g/L xylose to analyze and compare the performance of cell growth, xylose utilization, and ethanol production (Fig. 4). One significant difference was that except for the first transfer, all other transfers exhibited cell growth advantages over the control strain 8b. The more the transfer was conducted, the better the cell growth was obtained. As shown in Fig. 4a, the growth of the 15th, 19th, and 38th transfers had a significant advantage over the 1st, 8th, and 11th transfers. The 38th transfer, named as strain 8b-S38, achieved the maximum OD_600_ of 6.36 at the stationary phase (72 h), which was 2.02 folds higher than that of the parental strain 8b (OD_600_ of 3.15).

The improvement in xylose utilization and ethanol production was observed as well (Fig. 4b). With the increase of the numbers of transfers from 1st to 38th by ALE, the capability of the *Z. mobilis* recombinant strain 8b-*RsXI-xylB* in xylose consumption and ethanol production was increased. Strain 8b-S38 exhibited efficient xylose utilization capability, and can consume 50 g/L xylose in 42 h. While the unadapted control strain 8b only utilized 68% xylose at the end of the fermentation (Fig. 4b). Corresponding to the better xylose utilization, the maximum ethanol titer of 23.70 g/L was achieved by strain 8b-S38, which was 1.52-fold than that of the parental strain 8b. Therefore, strain 8b-S38 was further applied for xylose utilization evaluation (Fig. 4b).

### Fermentation performance evaluation of *Z. mobilis* 8b-S38 with glucose or xylose as the sole carbon source

To verify the capability of *Z. mobilis* strain 8b-S38 in xylose utilization, 50 g/L, 100 g/L, and 150 g/L xylose were selected to evaluate its fermentation performance in terms of growth, sugar utilization, and ethanol production. In general, all strains including 8b and the adapted strains grew significantly better in glucose medium with the average specific growth rate of 0.40 compared with those in xylose media that were not exceeded 0.12. In addition, strain 8b grew better than the other two adapted strains when glucose is the sole carbon source (Additional file 2: Table S1), which is consistent with previous results [17].

**Fig. 5:**
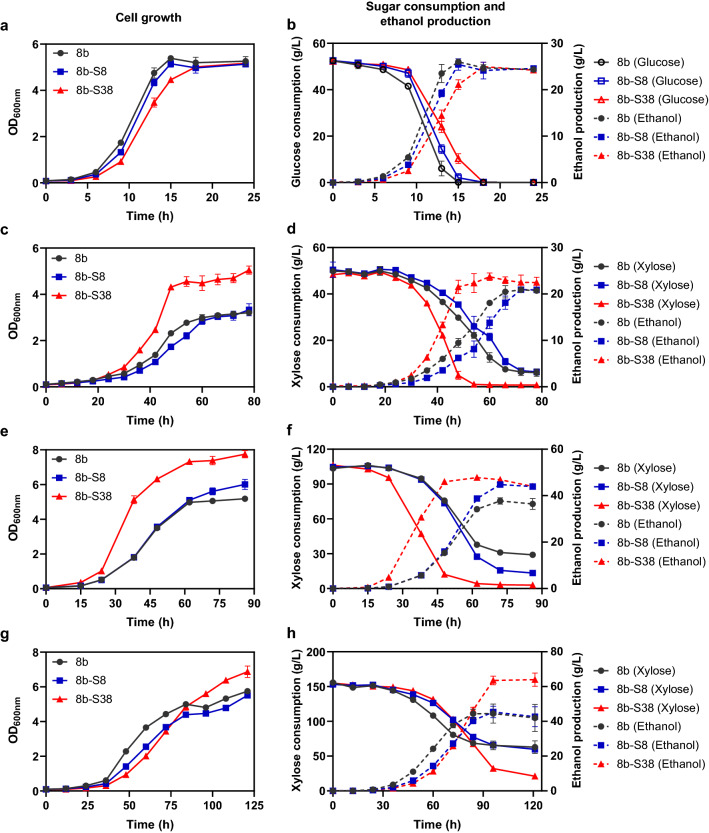
Cell growth, sugar consumption, and ethanol production of strain 8b, 8b-S8, and 8b-S38 in RMG5 (**a**, b), RMX5 (**c**, **d**), RMX10 (**e**, **f**), and RMX15 (**g**, **h**). Strains were cultured in 40 mL RMG or RMX in 50-mL shake flasks at 100 rpm and 30 °C. Spectinomycin at a final concentration of 50 μg/mL was added for recombinant strains. Three replicates were performed for each experiment. RMG5, RMX5, RMX10 and RMX15 represented RM media containing 50 g/L glucose, 50 g/L xylose, 100 g/L xylose, and 150 g/L xylose, respectively

The result suggested that sugar sources had a great effect on cell growth, and glucose was superior to xylose as the carbon source for ethanol fermentation. The slower consumption of xylose and less energy generation for cell growth may be the main reason for slower xylose metabolism as reported before [39]. As depicted, all strains could consume 50 g/L glucose completely to get a maximum OD_600_ around 5.20 and ethanol titer around 25 g/L (Fig. 5a, b). However, it took 8b-S38 strain 18 h to consume all glucose, while 8b utilized all glucose within 15 h (Fig. 5b). *Z. mobilis* 8b possessed a maximum ethanol productivity of 1.73 g/L/h, which was 1.24-fold of 8b-S38 (Additional file 2: Table S1). The result suggested that the increase of xylose utilization capability in strain 8b-S38 reduced its growth and the corresponding ethanol productivity when pure glucose was used as the carbon source (Additional file 2: Table S1).

When strains were cultured in xylose media, strain 8b-S38 exhibited a great growth advantage over strain 8b and 8b-S8, especially under 50 g/L and 100 g/L xylose (Fig. 5c–h). Under 50 g/L xylose, strain 8b-S38 achieved the final OD_600_ of 5.05, about 1.57 times and 1.51 times higher than that of 8b and 8b-S8, respectively (Fig. 5c). In addition, strain 8b-S38 consumed all xylose with a maximum ethanol titre of 23.72 g/L at 60 h, while the parental strain 8b only utilized 88% xylose with 20.46 g/L ethanol produced (Fig. 5d).

The superiority of strain 8b-S38 in xylose metabolism was further manifested when it was grown in 100 g/L xylose. The maximum OD_600_ of 7.75 in strain 8b-S38 was achieved at 86 h, about 1.50 times and 1.30 times higher than that of 8b and 8b-S8 (Fig. 5e). With the increase of the xylose concentration, more biomass can be obtained due to the provision of more carbon source. Notably, strain 8b-S38 nearly consumed all xylose within 72 h, while strain 8b and strain 8b-S8 only utilized 72% and 90% xylose, respectively. Correspondingly, the maximum ethanol titer of 47.78 g/L was achieved by strain 8b-S38 under 100 g/L xylose (Fig. 5f).

However, when the xylose concentration increased to 150 g/L, 8b-S38 did not exhibit growth advantage with similar growth rate as 8b and 8b-S8, although 8b-S38 had a higher maximum OD_600_ of 6.87 compared with 8b and 8b-S8 (Fig. 5g). Although all three strains could not utilize 150 g/L xylose completely, 8b-S38 still displayed a better xylose consumption capability with nearly 86% xylose consumed and 63.97 g/L ethanol produced (Fig. 5h).

Compared with the fermentation under 50 g/L xylose and 100 g/L xylose, the xylose consumption and ethanol production were decreased in all three strains under 150 g/L xylose. This phenomenon was consistent with previous works that the higher the xylose concentration is, the more difficult it is for cells to complete fermentation [12, 16, 39]. It can be attributed to the production of ethanol and toxic intermediates derived from xylose such as xylitol and xylonate [31, 40]. Higher ethanol can be produced under higher xylose concentration, which in turn inhibited xylose fermentation and resulted in less xylose consumption. Accumulations of toxic intermediates like xylitol or xylonate during the fermentation process will reduce the overall xylose metabolism efficiency and inhibit cell growth [14, 16, 31].

Fermentation performance under three different xylose conditions confirmed the efficient xylose utilization in strain 8b-S38 over the parental strain 8b and the intermediate strain 8b-S8. The capability of strain 8b-S38 in xylose utilization was further compared with other *Z. mobilis* strains that have been engineered and reported previously to be able to utilize xylose as the carbon source (Additional file 2: Table S2).

Strain AD50 is the recombinant strain with the highest xylose utilization capability reported till date exhibiting a maximum ethanol productivity of 1.02 g/L/h and comparable theoretical yield of ethanol (98%) [30]. In this study, strain 8b-S38 also possessed a superior xylose utilization capacity than other previously developed strains producing a similar amount of ethanol as that of AD50 (47.8 g/L vs 49 g/L), although the ethanol productivity of 8b-S38 under 100 g/L xylose was lower than that of AD50 (Additional file 2: Table S2).

Interestingly, all these xylose-utilizing strains listed in Additional file 2: Table S2 were developed at least partially using the ALE strategy, except for strain 31821 (pKLD4) that was constructed by metabolic engineering approach only, which had inferior xylose fermentation performance than other strains [14]. This result suggests that ALE is an effective strategy that can be combined with rational strain design and construction using metabolic engineering approach to help improve complex phenotypes such as efficient xylose utilization in this study.

### Evaluation of *Z. mobilis* 8b-S38 performance in mixed sugars of glucose and xylose

**Fig. 6:**
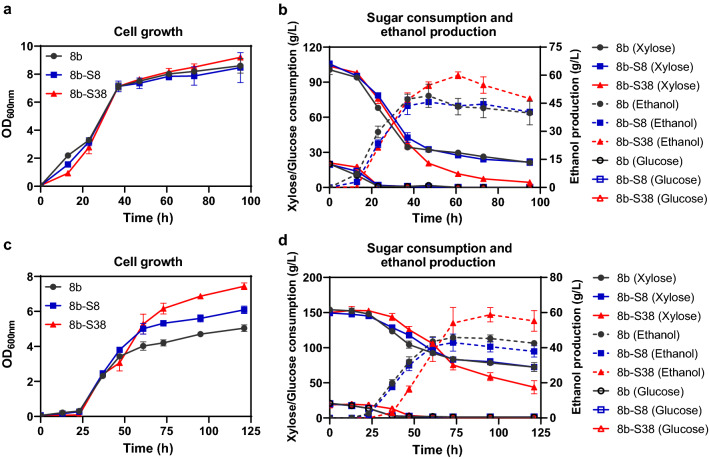
Cell growth, sugar consumption, and ethanol production of xylose-utilization strains 8b, 8b-S8, and 8b-S38 in mixed sugars of RMG2X10 (**a**, **b**) and RMG2X15 (**c**, **d**). Strains were cultured in 40 mL RMGX in 50-mL shake flasks at 100 rpm and 30 °C. Spectinomycin at a final concentration of 50 μg/mL was added for recombinant strains. Three replicates were performed for each experiment. RMG2X10 and RMG2X15 represented RM media containing different concentrations of mixed sugars of glucose and xylose. RMG2X10 contains 20 g/L glucose and 100 g/L xylose, RMG2X15 contains 20 g/L glucose and 150 g/L xylose

Two mixed-sugar media containing 20 g/L glucose and 100 g/L xylose (G2X10) or 20 g/L glucose and 150 g/L xylose (G2X15) were further used to evaluate the fermentation performance of 8b, 8b-S8, and 8b-S38 on cell growth, sugar utilization, and ethanol production (Fig. 6, Additional file 2: Table S3). Three strains grew similarly without significant differences in G2X10 media although 8b-S38 had a slight advantage over strain 8b and 8b-S8 with a final OD_600_ of 9.21 (Fig. 6a). All strains consumed glucose completely within 23 h. However, only strain 8b-S38 consumed almost all xylose at 95 h with a maximum consumption rate 1.77 g/L/h, while strain 8b and 8b-S8 only utilized 80% xylose. Correspondingly, strain 8b-S38 achieved the maximum ethanol titre of 59.92 g/L with the maximum productivity 0.98 g/L/h, about 1.38 times and 1.20 times higher than those of 8b and 8b-S8 (Fig. 6b, Additional file 2: Table S3).

The fermentation advantage of 8b-S38 in the mixed-sugar conditions was demonstrated as well when it was cultured in G2X15 media. As shown in Fig. 6c, the final OD_600_ of strain 8b-S38 was 7.43, about 1.50 times and 1.22 times higher than those of 8b and 8b-S8. Compared with the fermentation in G2X10 media, all glucose was utilized by three strains, but with a longer time of 37 ~ 47 h. Although all three strains could not utilize xylose completely, strain 8b-S38 still consumed xylose better than other two strains with more than 70% xylose utilized and 58.76 g/L ethanol achieved (Fig. 6d).

The capability of strain 8b-S38 in mixed-sugar utilization was further compared with other xylose-utilization strains of *Z. mobilis* reported before in literature. Despite that the ethanol yield of 8b-S38 in 100 g/L xylose media was lower than that of AD50 strain (Additional file 2: Table S2), its yield in mixed-sugar media was comparable with AD50 (96%) [30] in mixed sugars, with 97.58% in G2X10 and 92.85% in G2X15 media (Additional file 2: Table S3). Strain FR2 was another newly engineered strain for efficient xylose fermentation with another copy of *xylAB* and *talB*-*tktA* inserted in the genome of parental strain 8b and its ethanol yield in the mixed-sugar media was 95.5% [41]. Interestingly, another engineered strain FR1 was also constructed in that work with only *talB*-*tktA* inserted in 8b, but showed no difference to the parental strain. All these results suggested that overexpression of xylose isomerase and xylulokinase genes in 8b is important for efficient xylose utilization.

### Morphological changes of *Z. mobilis* 8b-S38 during adaptation

**Fig. 7:**
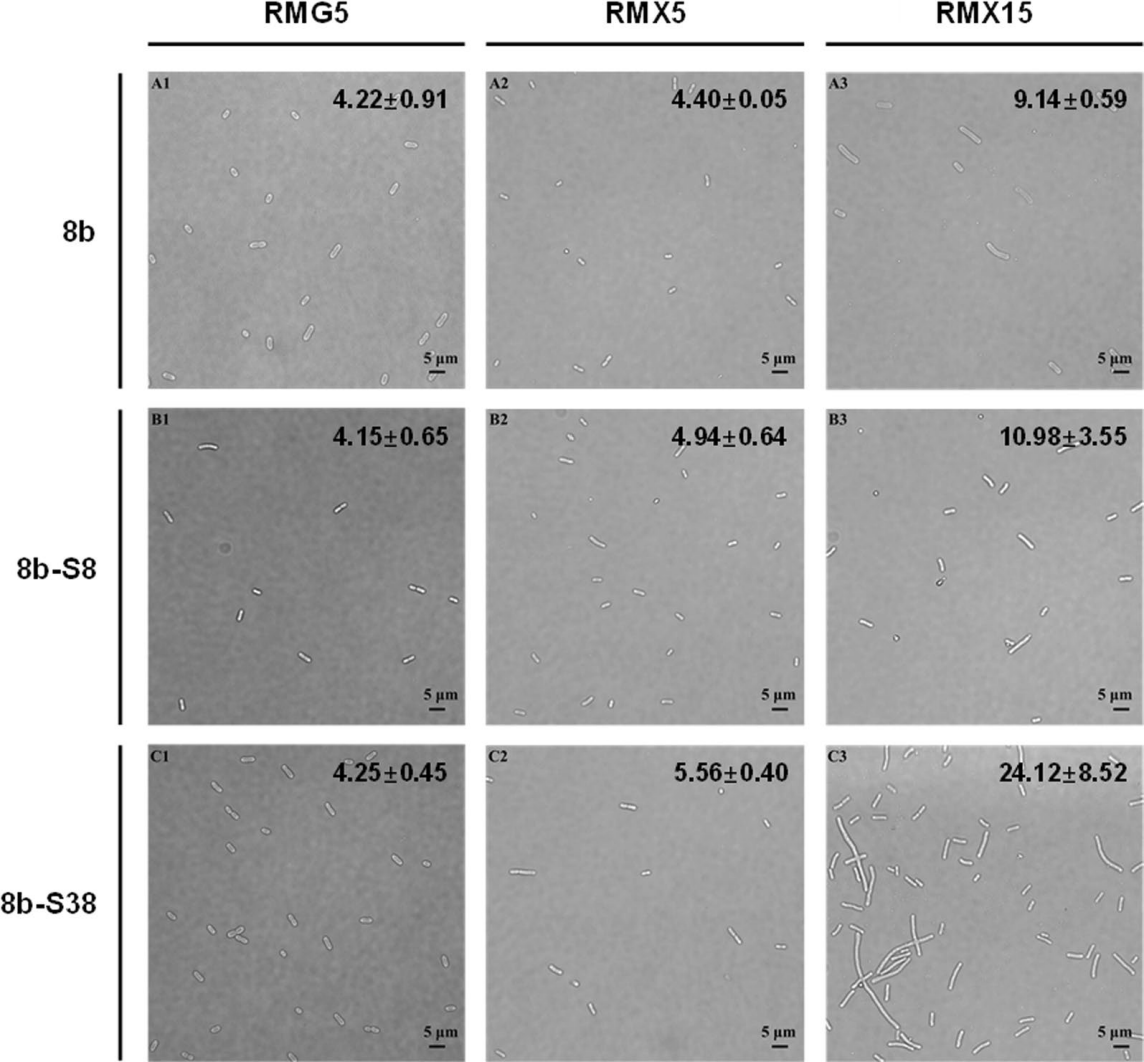
Cell morphology of strain 8b, 8b-S8, and 8b-S38 cultured in RMG5, RMX5, and RMX15 observed by light microscopy. Numbers in the upper right corner of each image represent the average cell size (μm) analyzed using the ImageJ software. RMG5, RMX5, and RMX15 represented the different media with 50 g/L glucose, 50 g/L xylose, 150 g/L xylose as the carbon source

To evaluate the morphological changes of *Z. mobilis* during the xylose adaptation and its relationship with the improved xylose utilization capacity, the cell morphologies of three *Z. mobilis* strains, 8b, 8b-S8, and 8b-S38 were observed by light microscopy. The result showed that all strains had normal short-rod shape with cell size around 4.0 ~ 5.0 μm in media containing 50 g/L glucose (Fig. 7). However, when strains were cultured in xylose as the carbon source, cells gradually changed from a short-rod shape to a filament one as the concentration of xylose increasing (Fig. 7). This result suggested that despite of improved xylose-utilization capability, adapted strains remained physiologically challenged by the xylose culture conditions, which could be due to the stressful environment of xylose media for *Z. mobilis* cells as reported before [42, 43]*.* Such morphological disturbances have been described previously in *Z. mobilis* when exposed to different stresses, such as high temperature [44] and inhibitory lignocellulosic hydrolysate [18].

Compared with the parental strain 8b, adapted strain 8b-S8 and 8b-S38 displayed cell size enlargement in xylose media, especially for strain 8b-S38. When cultured in 150 g/L xylose, the average cell size of 8b-S38 was 24.12 μm, which was 2.64 and 2.20 times of the size of 8b and 8b-S8, respectively (Fig. 7). However, the relationship between cell size and xylose utilization needs to be further investigated.

### Identification of genetic changes in *Z. mobilis* 8b-S38

To determine the underlying genetic determinants responsible for the enhanced xylose utilization and ethanol production in 8b-S38, we used next-generation sequencing (NGS) technology to identify the potential genetic changes in 8b-S38 and 8b-S8. The parental strain 8b was used as the reference strain for single-nucleotide polymorphism (SNP) characterization. No SNP was detected between strain 8b-S8 and 8b, which indicates that *Z. mobilis* is relatively genetically stable and eight transfers in xylose adaptation were not enough to generate stable mutations for efficient xylose utilization. Even after months of xylose adaption, there are only three stable SNPs identified in strain 8b-S38 compared with its parental strain 8b. The first two are non-synonymous SNPs located in gene *ZMO0578* and *ZMO0661*, encoding sodium:dicarboxylate symporter and chaperone protein DnaJ, respectively. The third is a synonymous one located in gene *ZMO0975* encoding a hypothetical membrane-spanning protein.

Sodium:dicarboxylate symporter encoded by *ZMO0578* is a member of the ubiquitous divalent anion/Na^+^ symporter (DASS) family which mediates the transport of C_4_‐dicarboxylates such as succinate or malate across the cell membrane typically by utilizing the pre-existing Na^+^ gradient [45]. Since succinate and other C_4_-dicarboxylates are important Krebs cycle intermediates which can be direct integrated into central metabolic pathways and served as good carbon and energy sources for growth [46], the mutation in *ZMO0578* in strain 8b-S38 might impact the uptake of these dicarboxylate and thus affect its cell growth in xylose media.

Chaperone protein DnaJ encoded by *ZMO0661* is a prototypical member of the heat shock protein (Hsp) Hsp40 family and functions as a co-chaperone of DnaK, the major bacterial Hsp70. As a co-chaperone, DnaJ enhances the ATPase activity of DnaK, synergistically with substrates. The main function of DnaJ and DnaK is known to be involved in the folding of newly synthesized or unfolded polypeptides [47]. Previous studies reported that DnaJ is involved in bacterial biofilm formation and affects cell viability and motility [48, 49]. The mutation in *ZMO0661* blocked the normal gene expression and led to the length of DnaJ protein from 375 amino acids to 206 amino acids. Further work is still needed to identify the role of DnaJ mutation in 8b-S38 on efficient xylose utilization, although we hypothesize that the potential mutation of DnaJ could reduce the ATPase activity of DnaK for energy conservation, which is consistent with our RNA-Seq result that gene encoding heat-shock protein repressor HrcA was induced compared with its parental strain 8b as discussed in details below.

### Transcriptional difference in *Z. mobilis* strains under different sugars conditions

To determine genes affected at the transcriptional level after adaption, RNA-Seq was employed to explore the global transcriptional differences in strain 8b-S38, 8b-S8, and 8b in different media. As a result, the differentially expressed genes (DEGs) were identified through analysis of variance (ANOVA) using strains and media as variables, and the results indicated that the difference between media is more dramatic than the difference among strains (Additional file 3: Table S4, Additional file 4: Table S5). The DEGs from comparisons of the strains in different sugar media or different strains in the media were then further analyzed.

#### Effect of different sugar media on *Z. mobilis* strains

The detailed gene expression information in response to the different sugar media in three strains is listed in Additional file 3: Table S4, and the number of differentially expressed genes is summarized in Additional file 5: Table S6. Consistent with previous studies [42, 43], the variable of sugar (glucose versus xylose) media caused dramatic transcriptional changes. When 50 g/L xylose was used as the sole carbon source, 643 genes were differentially expressed compared with that using glucose as the sole carbon source including 308 genes up-regulated and 335 genes down-regulated. When the xylose concentration increased to 150 g/L, more genes were differentially expressed, with 389 genes up-regulated and 405 genes down-regulated compared with RMG5 medium and 80 genes significantly differentially expressed compared with RMX5 medium. Apparently, the utilization of pentose sugar xylose posed a significant metabolic burden to *Z. mobilis* cells. The higher metabolic burden caused by the increase of xylose concentrations thus required more transcriptional regulation for cells to adapt, survive, and thrive.

The effects of different sugar media (glucose versus xylose) on three strains were further compared and analyzed. The results showed that xylose utilization involved in hundreds of genes differentially expressed in all three strains with more than 200 differentially up-regulated or down-regulated genes shared in three strains, which could be used to select genetic targets for improving xylose utilization in *Z. mobilis* in the future (Additional file 3: Table S4, Additional file 5: Table S6). This result also suggested that despite of improved xylose-utilization performance, 8b-S38 remained challenged by the xylose culture conditions, which was consistent with the cell morphology difference of these three strains in different media discussed above (Fig. 7).

#### Comparisons of different strains in different sugar media

The detailed gene expression information of different strains comparisons is listed in Additional file 4: Table S5, and the number of differentially expressed genes is summarized in Additional file 5: Table S7. Unlike the dramatic transcriptomic changes in response to different sugar media, less differentially expressed genes were observed among strains. Consistent with the fermentation performance analyses, strain 8b-S38 was obviously different from the parental strain 8b and the intermediate adapted strain 8b-S8 (Fig. 5), 8b-S38 displayed a larger amount of change in the transcriptional regulation, with 68 genes differentially expressed compared with 8b (Additional file 1: Figure S2), and 35 genes differentially expressed compared with 8b-S8. Meanwhile, only 3 genes were detected to be differentially regulated in the comparison between strain 8b-S8 and 8b. This can be attributed to the adaptive evolution process, in which 8b-S38 was selected after a longer evolution time and thus more genes were influenced and regulated at the transcriptional level.

To illustrate the mechanism underlying improved xylose utilization in strain 8b-S38, the DEGs from strains comparisons in the xylose media were further investigated, especially for the comparisons of strain 8b-S38 with 8b or 8b-S8, since few DEGs were detected between 8b-S8 and 8b (Additional file 4: Table S5, Additional file 5: Table S7). In RMX5 media, 123 genes were differentially expressed in 8b-S38 versus 8b including 86 genes up-regulated and 37 genes down-regulated, while in RMX15 media, 195 genes were differentially expressed including 82 genes up-regulated and 113 genes down-regulated. The result of more genes differentially expressed in RMX15 further illustrated that xylose in the media was a stress for *Z. mobilis* cells. A similar phenomenon was observed in the strain 8b-S38 versus 8b-S8, with 89 genes differentially expressed in RMX5 and 154 genes in RMX15. All these differentially expressed genes could play a role on efficient xylose utilization for strain 8b-S38, which are discussed below in details.

### Mechanism of efficient xylose utilization in *Z. mobilis* 8b-S38

#### Carbon metabolism

Gene expression in strain 8b-S38 showed that three genes involved in the pentose phosphate pathway and glycolysis pathway, *ZMO1200* (*rpiB*), *ZMO1212* (*pgi*), and *ZMO1240* (*gpmA*), were significantly up-regulated in the xylose media compared with 8b or 8b-S8 (Additional file 6: Table S8). Since these genes are essential for the xylose metabolism and the ED pathway in *Z. mobilis* for ethanol production, the up-regulation of these genes could contribute to the enhanced xylose consumption rate in 8b-S38 (Fig. 5), which in turn may help produce more ATP for cell growth and xylose stress tolerance.

In addition, the expression of *ZMO0976* (*xyrA*) encoding xylose reductase that catalyzes the xylose reduction to xylitol was significantly down-regulated in xylose media compared with glucose media in all three strains. The up-regulation of *xyrA* could lead to the accumulation of toxic xylitol, and is a primary bottleneck in xylose fermentation to ethanol [16]. Therefore, the down-regulation of *ZMO0976* in strain 8b-S38 compared with 8b or 8b-S8 in the xylose media, especially in RMX15 media could contribute to its efficient xylose utilization capability, which is consistent with previous study that the mutation in *ZMO0976* with reduced xylose reductase activity improved xylose utilization of *Z. mobilis* [30]. This result can be attributed to the evolution process with xylose as the adaptive selection pressure (Fig. 3), in which most of the xylose has been adapted to be metabolized via the introduced xylose isomerase pathway in 8b-S38 and only a small amount of xylose was reduced by xylose reductase.

#### Protein and DNA repair

*Z. mobilis* can regulate universal stress-response genes to protect macromolecules including proteins and DNA from the damage caused by the stressful environments [12, 42–44, 50]. Gene expression in three strains demonstrated that the transcriptional level of *ZMO0246* (*hslV*), *ZMO0247* (*hslU*), *ZMO0405* (*clpA*), *ZMO1424* (*clpB*), and *ZMO1704* (*lon2*) involved in protein remodeling and reactivation, as well as *ZMO1231* (*recJ*) and *ZMO1588* (*uvrA*) involved in DNA repair were significantly up-regulated in response to the variable of sugar (glucose versus xylose) media (Additional file 6: Table S8). These results demonstrated that it is necessary to enhance the expression of these proteins to protect protein and DNA from damage in xylose media for *Z. mobilis* cells.

Gene expression in strains comparisons further discovered that these universal stress-response genes discussed above were up-regulated in 8b-S38 compared with 8b or 8b-S8 in the xylose media, especially in RMX5. Moreover, the expression level of genes encoding two other protein modeling-related chaperone proteins ZMO1928 (GroES) and ZMO1929 (GroEL) altered similarly as these universal stress-response genes in 8b-S38 (Additional file 6: Table S8). These results suggested that the overexpression of universal stress-response genes in 8b-S38 may be one of the strategies for cells to deal with the damages caused by xylose stress, which may explain why 8b-S38 grew better with higher xylose consumption and ethanol production in xylose media. Interestingly, the only gene with non-synonymous mutation in 8b-S38 compared with 8b is *ZMO0661* (*dnaJ*), which is involved in protein folding as well [47]. Therefore, the regulation of universal stress-response genes is important for *Z. mobilis* to survive in xylose media.

#### Flagellar biosynthesis

Bacterial flagellum is a complex and dynamic nanomachine appended on the cell body that provides motility [51]. Recently, it was reported to be critically important in bacterial survival, reproduction, and pathogenicity, such as adhesion to a variety of substrates, secretion of virulence factors, and formation of biofilms [52]. Gene expression in RMX15 media compared with RMG5 media showed that 13 genes related to flagellar biosynthesis (such as *flgBCDEFGHI*, *flhA*, and *fliEFG*) were down-regulated in strain 8b and 8b-S8, while almost no differentially expressed flagellar-related genes were detected in the strain 8b-S38. Moreover, the gene expression in the strain comparisons showed that these 13 genes mentioned above were significantly up-regulated in 8b-S38 compared with 8b or 8b-S8 in the RMX15 media (Additional file 6: Table S8). All these results suggested that high concentration of xylose as the sole carbon source in the media could downregulate the energy-costly flagellar assembly process in strain 8b or 8b-S8 to help conserve energy from cell motility for survival in the xylose stressful condition as previously reported [12, 43], but had almost no effect on this process in strain 8b-S38. This can be attributed to the overexpression of these carbon metabolism-related genes with enough energy generation or these universal stress-response genes to effectively deal with the xylose stress as mentioned above, thus 8b-S38 didn't have to alter its flagellar synthesis process. On the other hand, the unaffected flagellar assembly process in strain 8b-S38 could maintain its normal cell locomotion in xylose media and thus actively search out better conditions, relief from the carbon catabolite repression or ‘foraging’-like behavior to utilize carbon sources [53], which would contribute to its improved xylose fermentation performance.

#### Phage shock protein response

The phage shock protein (Psp) response was initially found in *E. coli* infected by phages, and considered as a stress response of cells to the phages [54]. Now, Psp system was identified to perceive cell membrane stress and signal to the transcription apparatus by using an ATP hydrolytic transcription activator (PspF) to produce Psp effectors (PspA, PspD) to maintain and conserve the proton-motive force (PMF) under stress conditions, such as secretins, extremes of heat, ethanol, and osmolarity [55, 56]. Gene expression results showed that the expression level of genes encoding phage shock proteins including *ZMO1062* (*pspD*), *ZMO1063* (*pspA*), *ZMO1064* (*pspB*), and *ZMO1065* (*pspC*) were significantly up-regulated not only in three strains in xylose media compared with the glucose media, but also in 8b-S38 in the xylose media (RMX5) compared with 8b or 8b-S8 (Additional file 6: Table S8). The result indicated that the xylose might trigger stress pressure to the cell membrane, and 8b-S38 can manage the pressure by preventing proton leakage across the membranes and thus maintain the PMF and membrane permeability through regulating genes encoding Psp proteins.

#### Protein synthesis

Consistent with previous stress response transcriptomic studies in *Z. mobilis* [42–44], xylose as a stressor inhibited the cellular biosynthesis process with many biosynthesis genes down-regulated in the sugars comparison (xylose versus glucose) in all three strains (Additional file 5: Table S4). However, among these genes, 15 ribosomal protein-related genes (such as *rplO*, *rplQ*, *rplJ*, *rplL*, and *rpsI*) and 6 amino acid biosynthesis-related genes (*glnB*, *glnA*, *hisC3*, *glyA*, *hisG*, *hisD*) were significantly up-regulated more than twofold in strain 8b-S38 compared with 8b in RMX5 and RMX15 media (Additional file 6: Table S8). The up-regulation of about 20 genes involved in protein synthesis might contribute to the improved growth of 8b-S38 in xylose media.

#### Transcriptional regulation

Gene expression in strains comparisons showed that 10 transcriptional regulators including repressor HrcA (ZMO0015), YebC/PmpR family (ZMO0153), Fis family (ZMO0631), repressor Maf (ZMO1013), regulator NrdR (ZMO1202), LysR family (ZMO1206, ZMO1336), Fur family (ZMO1235), HxlR family (ZMO1697), and activator NifA (ZMO1816) were differentially expressed (Additional file 6: Table S8). Among these regulators, HrcA is a negative regulator protein of heat shock genes (*grpE*-*dnaK*-*dnaJ* and *groELS* operons) [57], which is significantly up-regulated in 8b-S38 in the xylose media (RMX5 and RMX15) compared with 8b or 8b-S8. The overexpression of HrcA might help rationally regulate these heat shock genes and redirect energy toward the increased expression of genes more directly involved in the protective responses to the xylose environment.

NrdR is a regulator protein that represses the transcription of genes encoding ribonucleotide reductases (RNRs) involved in de novo DNA synthesis and repair by catalyzing the conversion of ribonucleotides to deoxyribonucleotides [58]. Its differential expression in strain 8b-S38 in xylose media might be involved in protecting cells from the damage caused by the xylose environment through the DNA synthesis and repair process regulation. Interestingly, protein Maf is a repressor that binds to both ComGA and DivIVA, blocks cell division, and leads to the cells filamented slightly [59]. Therefore, the up-regulation of Maf in strain 8b-S38 might contribute to the morphological changes in xylose media as observed in Fig. 7.

**Fig. 8:**
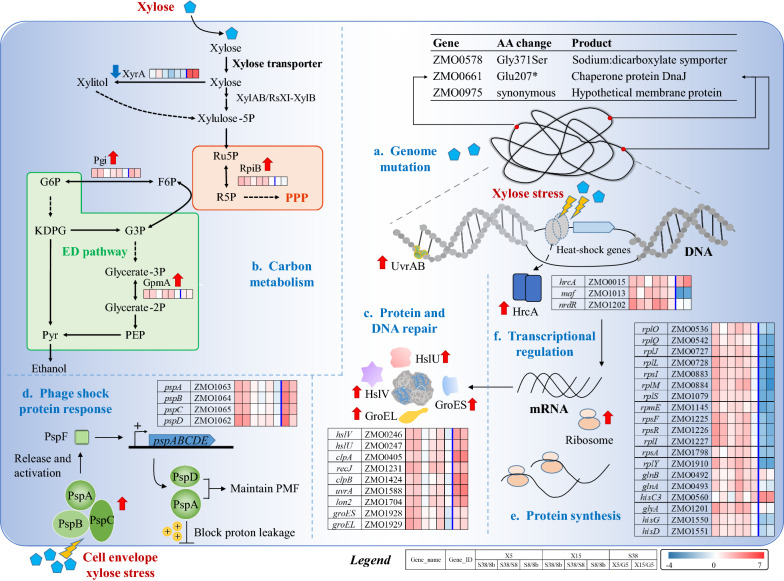
The potential mechanism contributing to the efficient xylose utilization of the adapted strain 8b-S38. Three mutations identified in strain 8b-S38 compared with the parental strain 8b (**a**); up-regulation of central carbon metabolism and down-regulation of xylitol production leading to more xylose metabolized via xylose isomerase (XI) pathway (**b**); up-regulation of protein and DNA repair system to deal with the damage caused by xylose stress (**c**); the phage shock protein response triggered to prevent proton leakage thus to maintain the proton-motive force (PMF) and membrane permeability (**d**); up-regulation of the protein synthesis process contributing to the cell growth (**e**); differentially expressed genes related to several transcriptional regulators (**f**). The red up or blue down arrow beside the enzyme represents up-regulation and down-regulation, respectively. The details of gene expression are shown in the Additional file 6: Table S8

Although the transcriptional changes associated with the adapted strain 8b-S38 as discussed above suggested that the transcriptional changes of the adapted strain 8b-S38 inherited during the adaptation may affect the expression of genes involved in carbon utilization, protein synthesis, general stress responses, as well as cell morphology and motility contribute to the efficient xylose-utilization phenotype of the adapted strain 8b-S38 (Fig. 8), further investigation of the roles of these transcriptional regulators and their interactions in 8b-S38 for efficient xylose utilization is needed.

## Conclusion

In summary, various recombinant strains for xylose utilization were constructed in *Z. mobilis*. The results indicated that xylose isomerase genes of *PiXI*, *RsXI*, and xylulokinase gene *xylB* combined with a complete PPP can enable *Z. mobilis* to utilize xylose. Furthermore, the increase of a copy of xylose isomerase and xylulokinase genes in strain 8b proved to be effective strategy for efficient xylose utilization. Strain 8b-RsXI-xylB had the highest growth in xylose media compared with other two recombinant strains, which was then subjected to continuous adaptation in xylose media to improve xylose utilization capability. Of which, 8b-S38 was the best among these mutants that can efficiently metabolize xylose for ethanol production at high xylose concentrations as well as mixed sugars of glucose and xylose. Fermentation of 8b-S38 under different xylose concentrations further verified its potential in xylose utilization and ethanol production, which could be used as the microbial biocatalyst for the production of cellulosic biofuels and biochemicals. Our work thus demonstrated that the introduction of additional xylose isomerase and xylulokinase can enhance xylose utilization.

In addition, this study also suggested the necessity to combine the approaches of metabolic engineering and adaptive laboratory evolution for industrial strain development, which help generate a series of mutant strains of *Z. mobilis* that can efficiently utilize xylose. Combining NGS-based genome resequencing and RNA-Seq, we also revealed that the genome of *Z. mobilis* is relatively stable, and genes involved in different cellular functions such as carbon metabolism, macromolecules repair, protein synthesis, and flagellar assembly contributed to the efficient xylose utilization. The molecular mechanism of efficient xylose utilization provides genetic candidates and rational design principles for subsequent genetic engineering and the construction of synthetic cell factories for efficient xylose utilization in the future.

## Materials and methods

### Strains, media, and culture conditions

Bacterial strains and plasmids used in this study are listed in Additional file 7: Table S9. *Z. mobilis* ZM4 (ATCC 31821) and its xylose-engineered strain 8b [13] were used as the parental strains for genetic modifications in this study. Where indicated, *Z. mobilis* strains were cultured at 30 °C with shaking at 100 rpm in Rich Medium (RM, 10 g/L yeast extract, 2 g/L KH_2_PO_4_, different concentrations of glucose and/or xylose as required, and 1.5% agar for solid). *Escherichia coli* DH5α was used for plasmid construction. All *E. coli* strains were cultured at 37 °C, 250 rpm, in Luria–Bertani medium (LB, 10 g/L NaCl, 10 g/L tryptone, 5 g/L yeast extract, and 1.5% agar for solid). The recombinant strains of *E. coli* and *Z. mobilis* were supplemented with 100 μg/mL spectinomycin. Different concentrations of tetracycline for induction were added in RM for *Z. mobilis* strains as needed.

### Genetic manipulation and recombinant strains construction

Three different xylose isomerases genes, *PiXI* from *Piromyces* sp. E2 [32], *RsXI* (*Rs-XI-N337C*) from the protists in the *Reticulitermes speratus* hindgut [33], and *RuXI* (*Ru-XI-K11T/D220V*) from bovine rumen [34] were synthesized by Genscript (Nanjing, China). After codon optimization, the gene was cloned into the minimized shuttle plasmid pEZ15Asp with P*tet* as the promoter [10] and three recombinant plasmids pEZ-*Pi*, pEZ-*Rs*, and pEZ-*Ru* were obtained, respectively. Xylose isomerase gene *xylB* was amplified from the genomic DNA of *E. coli* K-12. After purification, the gene was inserted after the XI gene and expressed as an operon to construct plasmids of pEZ-*PiXI-xylB*, pEZ-*RSXI-xylB*, and pEZ-*RuXI-xylB*.

All plasmids were assembled by the Gibson assembly method [60]. Briefly, primers were designed to contain 15 ~ 25 nucleotides overlapping regions with adjacent DNA fragments. PCR products amplified by primer pairs were purified and quantified using NanoDrop 2000 (Thermo Fisher Scientific, USA). Fragment and vector were mixed in a molar ratio of 3:1, 0.5 U T5 exonuclease (NEB, USA), 0.5 μL buffer 4 (NEB, USA), and ddH_2_O were added in a final volume of 5 μL. All reagents were mixed and incubated on ice for 5 min, and then added to chemically competent *E. coli* cells. After incubated on ice for 30 min, heat-shocked for 45 s at 42 °C, and held on ice for 2 min, 100 μL of NZY medium (5.0 g/L yeast extract, 5 g/L NaCl, 1.2 g/L MgCl_2_, 1.5 g/L MgSO_4_, 3.6 g/L glucose, 10 g/L casein enzymatic hydrolysate NZ amine®) was added to the mixture above and recovered for at least 1 h at 37 °C with shaking (250 rpm). Cells were plated on LB agar plates containing spectinomycin and recombinants were selected by colony PCR and confirmed by Sanger sequencing (TsingKe Biological Technology, Beijing, China).

The correct recombinant plasmids were transformed into *Z. mobilis* ZM4 or 8b competent cells, which were prepared as described previously [10], via electroporation (0.1-cm electrode gap, 1600 V, 200 Ω, 25 μF) using a Gene Pulser® (Bio-Rad, USA). Colonies with correct PCR product size were selected as candidate strains.

### Adaptive laboratory evolution (ALE)

RMX5 containing 50 g/L xylose as the sole carbon source was used to evolve *Z. mobilis* 8b-*RsXI*-*xylB*. Cells were firstly streaked on RMG2 plate, then three colonies were selected randomly and inoculated in liquid RMG2. After grown to the exponential phase, 100 μL of the culture was transferred to a tube containing 5 mL of fresh RMX5 medium to begin the adaptive laboratory evolution. When the strain was grown to the exponential phase, 100 μL was transferred to the new medium for the next round of adaptive evolution. In the first 1–3 rounds, the strain grew poorly in RMX5, so the strain was transferred to RMG2X5 for resuscitation in the 4th-5th rounds. After the growth was restored, the strain was transferred to RMX5 for adaptive evolution again. Finally, the strain with enhanced and stable growth in RMX5 was obtained until the 38th transfer.

### Growth curve measurement of engineered strains using Bioscreen C

Cell growth of different engineered *Z. mobilis* strains was monitored by measuring the cell OD values using a Bioscreen C high-throughput growth measurement instrument (Growth Curves USA, NJ, USA) with three technical replicates [10]. *Z. mobilis* strains were firstly revived from frozen glycerol stocks overnight without shaking in 10 mL of RMG2 to the exponential phase as the seed culture. The seed culture was washed twice then transferred into RMX5 with an initial OD_600 nm_ value of 0.1. Cells were inoculated into Bioscreen C wells containing a total volume of 300 µL and incubated without shaking at 30 °C. Cell growth was monitored automatically at 600 nm at regular intervals of 15 min. The experiments were repeated at least twice.

### Batch fermentation analysis in shake flasks

Seed cultures of *Z. mobilis* strains were harvested at exponential phase then inoculated into 50-mL shake flasks with 40 mL medium with an initial OD_600 nm_ value of 0.1. The cultures were maintained at 30 °C with an agitation rate of 100 rpm. During the fermentation, cell growth was determined at different time points by measuring the optical density at 600 nm (OD_600_) using an ultraviolet spectrophotometer (UV-1800, AOE, China). The samples were also collected and centrifuged at 13,680 × *g* for 2 min, and filtered through 0.22-μm filters. Samples at the exponential phase were harvested for genome resequencing and RNA-Seq. Glucose, xylose, and ethanol in the filtered supernatants were determined using high-performance liquid chromatography (HPLC, LC-20 AD, refractive index detector RID-10A, Shimadzu, Kyoto, Japan) with an Aminex HPX-87H column (Bio-Rad, Hercules, CA, USA) at 60 °C. Dilute sulfuric acid (5 mM) was used as the isocratic mobile phase at a flow rate of 0.5 mL/ min.

### Cell morphology observation and determination of cell size

*Z. mobilis* strains cultured in different conditions were collected at the exponential phase, washed twice with phosphate buffer saline (PBS), resuspended in the same buffer, and observed under a microscope (Leica DMi8, Illinois, USA) at × 400 magnification. Each image was taken using the image-based autofocus system LAS X software of the Leica DMi8 system, and the cell size was measured using the ImageJ software.

### Genome resequencing analysis and RNA-Seq transcriptomic analysis

Collected samples were entrusted to GENEWIZ (Suzhou, China) to complete the genome and transcriptome sequencing. The samples were evaluated for sample quality after DNA or RNA extraction and purification. When the quality was qualified, the library was built, and the Next-Generation Sequencing (NGS) technology was used to obtain the sequence data. Genome resequencing was performed based on the Illumina MiSeq sequencing platform by the paired-end sequencing technology according to standard Illumina protocols. The paired-end reads quality was checked using FastQC program (http://www.bioinformatics.babraham.ac.uk/projects/fastqc/). Data that passed the quality control were then mapped to the reference genome of *Z. mobilis* ZM8b (GenBank accession No. of chromosome: NZ_CP023682.1, and plasmids: NZ_CP023683.1, NZ_CP023684.1, NZ_CP023685.1, NZ_CP023686.1) using the CLC Genomics Workbench (version 14.0) to identify the genomic variations, in which the SNP frequency of 35%, minimal coverage cutoff of 10, and read count of 2 were set for SNP calling. The objective mutations in the mutant strain were obtained after removing the same mutations both in the mutant and the parental strains.

Transcriptome data were sequenced based on the Illumina HiSeq 2000 sequencer by the paired-end sequencing technology to obtain offline sequence data. RNA-Seq fastq data passed the quality control evaluated by FastQC program were imported into CLC Genomics Workbench (version 14.0) for reads trimming and RNA-Seq analysis to get the RPKM value (reads mapping to the genome per kilobase of transcript per million reads sequenced) of each gene with *Z. mobilis* ZM8b (GenBank accession No. of chromosome: NZ_CP023682.1, and plasmids: NZ_CP023683.1, NZ_CP023684.1, NZ_CP023685.1, NZ_CP023686.1) as the reference genome. Gene expression normalization, analysis of variance (ANOVA), and hierarchical clustering analysis were conducted using JMP Genomics (version 9.0) to identify differentially expressed genes at different conditions. Significantly differentially expressed genes were determined with a selection threshold of *P*-value ≤ 0.05 and log_2_-fold change ≥ 1 (significant induction) or ≤  − 1 (significant repression). Raw data were log_2_-transformed and imported. Duplicate samples were used for each condition.

## Supplementary Information

**Additional file 1: Figure S1.** Flowchart of the strain construction process for xylose utilization in *Z. mobilis*. **Figure S2.** Predicted interactions based on String 9.1 database for 36 up-regulated (**A**) and 32 down-regulated (**B**) genes in strain 8b-S38 compared with 8b with at least twofold changes from RNA-Seq transcriptomic study.

**Additional file 2: Table S1.** Comparisons of fermentation performance of xylose-utilization strains of 8b, 8b-S8, and 8b-S38 in media with different concentrations of glucose or xylose. **Table S2.** Comparison of the capability of different recombinant strains of *Z. mobilis *in xylose utilization and ethanol production. **Table S3.** Comparisons of fermentation performance of 8b strains in media with mixed glucose (G) and xylose (X).

**Additional file 3: Table S4.** List of all the significantly differentially expressed genes in the three strains in response to the different sugar media.

**Additional file 4: Table S5**. List of all the significantly differentially expressed genes of different strains comparison in different sugar media.

**Additional file 5: Table S6.** Summary of the numbers of significantly differentially expressed genes in different media.** Table S7.** Summary of the numbers of significantly differentially expressed genes among different strains in different media.

**Additional file 6: Table S8.** List of significantly differentially expressed genes in different functional categories comparing different strains in the same media or same strain cultured in different sugar media.

**Additional file 7: Table S9.** Plasmid and strains used in this study.

## Data Availability

The raw data of genome resequencing and RNA-Seq were deposited into Sequence Read Archive (SRA) database with the BioProject accession numbers of PRJNA749177 and PRJNA575654, respectively.
