# Supplementary material for: Development and characterization of efficient xylose utilization strains of Zymomonas mobilis
Source: Biotechnol Biofuels. 2021 Dec 4;14:231. doi: 10.1186/s13068-021-02082-x (PMC8645129; doi:10.1186/s13068-021-02082-x)
Supplement: Supplementary file 1 — Additional file 1: Figure S1. Flowchart of the strain construction process for xylose utilization in Z. mobilis. Figure S2. Predicted interactions based on String 9.1 database for 36 up-regulated (A) and 32 down-regulated (B) genes in strain 8b-S38 compared with 8b with at least twofold changes from RNA-Seq transcriptomic study. [file 13068_2021_2082_MOESM1_ESM.docx]

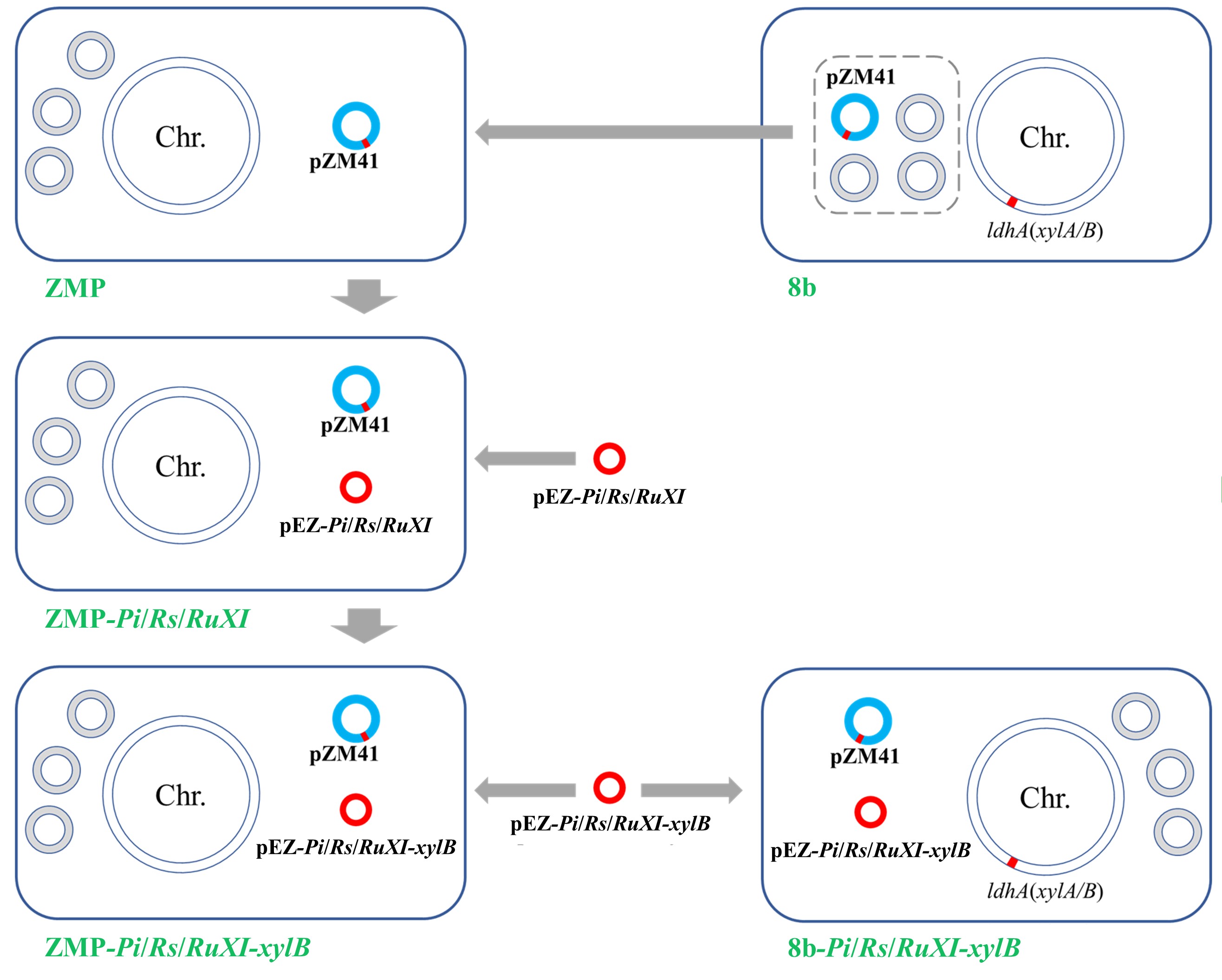


**Additional file 1:** **Figure S1.** Flow chart of the strain construction process for xylose utilization in *Z. mobilis*.

**Additional file 1: Figure S2.** Predicted interactions based on String 9.1 database for 36 upregulated (**A**) and 32 down-regulated (**B**) genes in strain 8b-S38 compared with 8b with at least 2-fold changes from RNA-Seq transcriptomic study.
