# Supplementary material for: Development and characterization of efficient xylose utilization strains of Zymomonas mobilis
Source: Biotechnol Biofuels. 2021 Dec 4;14:231. doi: 10.1186/s13068-021-02082-x (PMC8645129; doi:10.1186/s13068-021-02082-x)
Supplement: Supplementary file 2 — Additional file 2: Table S1. Comparisons of fermentation performance of xylose-utilization strains of 8b, 8b-S8, and 8b-S38 in media with different concentrations of glucose or xylose. Table S2. Comparison of the capability of different recombinant strains of Z. mobilis in xylose utilization and ethanol production. Table S3. Comparisons of fermentation performance of 8b strains in media with mixed glucose (G) and xylose (X). [file 13068_2021_2082_MOESM2_ESM.docx]

**Additional file 2: Table S1. Comparisons of fermentation performance of xylose-utilization strains of 8b, 8b-S8, and 8b-S38 in media with different concentrations of glucose or xylose.**

| **Medium** | **Strains** | **Specific**  **growth rate (/h)** | **Sugar**  **consumption**  **(g/L)** | **Max. sugar**  **consumption rates (g/L/h)** | **Ethanol**  **titer** **(g/L)** | **Ethanol**  **yield (%)** | **Max. ethanol**  **productivity**  **(g/L/h)** |
| --- | --- | --- | --- | --- | --- | --- | --- |
| **Glucose**  **50 g/L** | 8b | 0.42±0.01 | 52.39±0.65 | 3.57±0.24 | 24.91±1.90 | 93.31±8.20 | 1.73±0.01 |
|  | 8b-S8 | 0.41±0.00 | 52.41±0.43 | 3.36±0.15 | 25.44±1.23 | 92.99±1.83 | 1.70±0.08 |
|  | 8b-S38 | 0.38±0.01 | 52.17±0.21 | 2.89±0.01 | 24.97±0.95 | 93.82±3.29 | 1.39±0.05 |
| **Xylose**  **50 g/L** | 8b | 0.06±0.01 | 43.76±1.62 | 0.64±0.01 | 20.46±1.33 | 91.60±2.93 | 0.31±0.02 |
|  | 8b-S8 | 0.06±0.01 | 43.99±2. 02 | 0.60±0.03 | 19.85±1.85 | 95.62±1.53 | 0.29±0.01 |
|  | 8b-S38 | 0.09±0.01 | 47.60±0.68 | 0.79±0.01 | 23.72±0.81 | 97.68±2.01 | 0.40±0.01 |
| **Xylose**  **100 g/L** | 8b | 0.09±0.01 | 74.96±1.75 | 1.01±0.01 | 37.71±1.38 | 97.95±1.31 | 0.52±0.02 |
|  | 8b-S8 | 0.09±0.01 | 93.93±3.13 | 1.24±0.01 | 44.78±0.20 | 95.42±0.14 | 0.62±0.01 |
|  | 8b-S38 | 0.12±0.01 | 103.18±2.08 | 1.64±0.04 | 47.78±0.99 | 92.59±1.94 | 0.77±0.02 |
| **Xylose**  **150 g/L** | 8b | 0.08±0.01 | 92.86±7.49 | 0.94±0.04 | 44.45±5.51 | 93.71±6.31 | 0.46±0.06 |
|  | 8b-S8 | 0.08±0.01 | 93.37±2.18 | 0.91±0.03 | 45.39±3.28 | 95.26±4.77 | 0.47±0.03 |
|  | 8b-S38 | 0.07±0.01 | 133.92±5.07 | 1.24±0.05 | 63.97±3.78 | 93.64±3.95 | 0.53±0.03 |

**Additional file 2: Table S2. Comparison of the capability of different recombinant strains of *Z. mobilis* in xylose utilization and ethanol production.**

| **Strain** | **Xylose (g/L)** | **Max. xylose consumption rate (g/L/h)** | **Ethanol titer (g/L)** | **Max. ethanol productivity (g/L/h)** | **Ethanol yield**  **(%)** | **Reference** |
| --- | --- | --- | --- | --- | --- | --- |
| **A3** | 100 | - | 43.1 | - | 88.2 | [[1](#_ENREF_16)] |
| **31821(pKLD4)** | 50 | 0.48 | 16.9 | 0.17 | 72.6 | [[2](#_ENREF_14)] |
|  | 100 | 0.70 | 21.8 | 0.18 | 78.4 |  |
| **KLD1** | 100 | - | 30.0 | - | 58.8 | [[3](#_ENREF_15)]* |
| **AD50** | 100 | - | 49.0 | 1.02 | 98.0 | [[4](#_ENREF_30)] |
| **8b-S38** | 50 | 0.79 | 23.7 | 0.40 | 96.5 | This study |
|  | 100 | 1.64 | 47.8 | 0.77 | 90.8 |  |

*: data estimated from the figures in this reference.

1. Agrawal M, Mao Z, Chen RR: Adaptation yields a highly efficient xylose-fermenting *Zymomonas mobilis* strain. Biotechnol Bioeng 2011, 108(4):777-785.

2. Dunn KL, Rao CV: Expression of a xylose-specific transporter improves ethanol production by metabolically engineered *Zymomonas mobilis*. Appl Microbiol Biotechnol 2014, 98(15):6897-6905.

3. Dunn KL, Rao CV: High-throughput sequencing reveals adaptation-induced mutations in pentose-fermenting strains of *Zymomonas mobilis*. Biotechnol Bioeng 2015, 112(11):2228-2240.

4. Sarkar P, Mukherjee M, Goswami G, Das D: Adaptive laboratory evolution induced novel mutations in *Zymomonas mobilis* ATCC ZW658: a potential platform for co-utilization of glucose and xylose. J Ind Microbiol Biotechnol 2020, 47(3):329-341.

**Additional file 2: Table S3. Comparisons of fermentation performance of 8b strains in media with mixed glucose (G) and xylose (X).**

| **Medium** | **Strains** | **Specific**  **growth rate**  **(/h)** | **Sugar**  **consumption**  **(g/L)** | **Max. sugar**  **consumption rate (g/L/h)** | **Ethanol**  **titer** **(g/L)** | **Ethanol**  **yield (%)** | **Max. ethanol**  **productivity**  **(g/L/h)** |
| --- | --- | --- | --- | --- | --- | --- | --- |
| **Xylose**  **100 g/L + Glucose**  **20 g/L** | 8b | 0.13±0.01 | 79.33±6.43（X） | 1.46±0.07（X） | 48.95±6.23 | 86.04±1.05 | 0.71±0.05 |
|  |  |  | 20.00±0.01（G） | 0.82±0.02（G） |  |  |  |
|  | 8b-S8 | 0.13±0.01 | 84.00±3.61（X） | 1.69±0.02（X） | 50.28±9.44 | 94.03±1.33 | 0.82±0.15 |
|  |  |  | 19.73±0.46（G） | 0.78±0.04（G） |  |  |  |
|  | 8b-S38 | 0.13±0.01 | 99.33±0.58（X） | 1.77±0.04（X） | 59.92±1.85 | 97.58±2.55 | 0.98±0.03 |
|  |  |  | 21.07±1.85（G） | 0.85±0.08（G） |  |  |  |
| **Xylose**  **150 g/L + Glucose**  **20 g/L** | 8b | 0.14±0.01 | 80.33±5.13（X） | 0.95±0.01（X） | 45.83±0.88 | 89.89±3.28 | 0.63±0.01 |
|  |  |  | 19.76±1.09（G） | 0.49±0.03（G） |  |  |  |
|  | 8b-S8 | 0.16±0.01 | 77.00±2.00（X） | 0.91±0.03（X） | 44.51±5.61 | 90.86±9.26 | 0.61±0.08 |
|  |  |  | 18.81±0.41（G） | 0.47±0.01（G） |  |  |  |
|  | 8b-S38 | 0.24±0.01 | 106.3±12.0（X） | 1.02±0.09（X） | 58.76±4.07 | 92.85±5.51 | 0.80±0.06 |
|  |  |  | 18.01±0.41（G） | 0.41±0.01（G） |  |  |  |
