# Supplementary material for: Development and characterization of efficient xylose utilization strains of Zymomonas mobilis
Source: Biotechnol Biofuels. 2021 Dec 4;14:231. doi: 10.1186/s13068-021-02082-x (PMC8645129; doi:10.1186/s13068-021-02082-x)
Supplement: Supplementary file 5 — Additional file 5: Table S6. Summary of the numbers of significantly differentially expressed genes in different media. Table S7. Summary of the numbers of significantly differentially expressed genes among different strains in different media. [file 13068_2021_2082_MOESM5_ESM.docx]

**Additional file 2: Table S6.** Summary of the numbers of significantly differentially expressed genes in different media.

|  | **cGene** | **pGene** | **Total** | **Sum** | |
| --- | --- | --- | --- | --- | --- |
| **RMX5/RMG5 regulated genes** | | | | | |
| X5/G5_Up | 262 | 46 | 308 | 643 | |
| X5/G5_Down | 332 | 3 | 335 |  |  |
| 8b_X5/G5_Up | 250 | 40 | 290 | 639 | |
| 8b_X5/G5_Down | 342 | 7 | 349 |  |  |
| S38_X5/G5_Up | 289 | 68 | 357 | 715 | |
| S38_X5/G5_Down | 358 | 0 | 358 |  |  |
| S8_X5/G5_Up | 237 | 40 | 277 | 594 | |
| S8_X5/G5_Down | 310 | 7 | 317 |  |  |
| **RMX15/RMG5 regulated genes** | | | | |  |
| X15/G5_Up | 314 | 75 | 389 | 794 | |
| X15/G5_Down | 403 | 2 | 405 |  |  |
| 8b_X15/G5_Up | 319 | 74 | 393 | 827 | |
| 8b_X15/G5_Down | 426 | 8 | 434 |  |  |
| S38_X15/G5_Up | 313 | 82 | 395 | 793 | |
| S38_X15/G5_Down | 398 | 0 | 398 |  |  |
| S8_X15/G5_Up | 324 | 74 | 398 | 842 | |
| S8_X15/G5_Down | 439 | 5 | 444 |  |  |
| **RMX15/RMX5 regulated genes** | | | | |  |
| X15/X5_Up | 34 | 13 | 47 | 80 | |
| X15/X5_Down | 33 | 0 | 33 |  |  |
| 8b_X15/X5_Up | 42 | 14 | 56 | 103 | |
| 8b_X15/X5_Down | 47 | 0 | 47 |  |  |
| S38_X15/X5_Up | 33 | 9 | 42 | 84 | |
| S38_X15/X5_Down | 41 | 1 | 42 |  |  |
| S8_X15/X5_Up | 68 | 37 | 105 | 195 | |
| S8_X15/X5_Down | 89 | 1 | 90 |  |  |

Note: cGene: number of chromosome genes; pGene: number of plasmid genes; Total: total number of up-regulated or down-regulated genes, including cGene and pGene; Sum: the sum of total up-regulated and down-regulated genes.

**Additional file 2: Table S7.** Summary of the numbers of significantly differentially expressed genes among different strains in different media.

|  | **cGene** | **pGene** | **Total** | **Sum** |
| --- | --- | --- | --- | --- |
| **S38/8b regulated genes** | | | | |
| S38/8b_Up | 34 | 2 | 36 | 68 |
| S38/8b_Down | 31 | 1 | 32 |  |
| G5_S38/8b_Up | 67 | 2 | 69 | 136 |
| G5_S38/8b_Down | 62 | 5 | 67 |  |
| X5_S38/8b_Up | 79 | 7 | 86 | 123 |
| X5_S38/8b_Down | 36 | 1 | 37 |  |
| X15_S38/8b_Up | 70 | 12 | 82 | 195 |
| X15_S38/8b_Down | 102 | 11 | 113 |  |
| **S38/S8 regulated genes** | | | | |
| S38/S8_Up | 19 | 3 | 22 | 35 |
| S38/S8_Down | 12 | 1 | 13 |  |
| G5_S38/S8_Up | 13 | 4 | 17 | 37 |
| G5_S38/S8_Down | 19 | 1 | 20 |  |
| X5_S38/S8_Up | 67 | 7 | 74 | 89 |
| X5_S38/S8_Down | 15 | 0 | 15 |  |
| X15_S38/S8_Up | 57 | 9 | 66 | 154 |
| X15_S38/S8_Down | 84 | 4 | 88 |  |
| **S8/8b regulated genes** | | | | |
| S8/8b_Up | 0 | 0 | 0 | 3 |
| S8/8b_Down | 3 | 0 | 3 |  |
| G5_S8/8b_Up | 15 | 0 | 15 | 29 |
| G5_S8/8b_Down | 14 | 0 | 14 |  |
| X5_S8/8b_Up | 5 | 0 | 5 | 7 |
| X5_S8/8b_Down | 1 | 1 | 2 |  |
| X15_S8/8b_Up | 7 | 0 | 7 | 35 |
| X15_S8/8b_Down | 24 | 4 | 28 |  |

Note: cGene: number of chromosome genes; pGene: number of plasmid genes; Total: total number of up-regulated or down-regulated genes, including cGene and pGene; Sum: the sum of total up-regulated and down-regulated genes.
