# Supplementary material for: Development and characterization of efficient xylose utilization strains of Zymomonas mobilis
Source: Biotechnol Biofuels. 2021 Dec 4;14:231. doi: 10.1186/s13068-021-02082-x (PMC8645129; doi:10.1186/s13068-021-02082-x)
Supplement: Supplementary file 7 — Additional file 7: Table S9. Plasmid and strains used in this study. [file 13068_2021_2082_MOESM7_ESM.docx]

**Additional file 2: Table S9.** Plasmid and strains used in this study.

| **Plasmids/Strains** | **Description** | **Reference** |
| --- | --- | --- |
| **Plasmids** | | |
| pEZ15Asp | Shuttle vector contains *Z. mobilis* origin and *E. coli* origin p15A; Sp^R^; Biobrick-compatible | [[1](#_ENREF_1)] |
| pZM41 | *Z. mobilis* 8b plasmid containing *Tal*, *Tkt*, Cm^R^ | [[2](#_ENREF_2)] |
| pEZ-*EGFP* | pEZ containing codon-optimized *EGFP* genes driven by inducible promoter P*tet* | Lab stock |
| pEZ-*PiXI* | pEZ containing codon-optimized *PiXI* genes by inducible promoter P*tet* | This work |
| pEZ-*RsXI* | pEZ containing codon-optimized *RsXI* genes by inducible promoter P*tet* | This work |
| pEZ-*RuXI* | pEZ containing codon-optimized *RuXI* genes by inducible promoter P*tet* | This work |
| pEZ-*PiXI*-*xylB* | pEZ containing codon-optimized *PiXI* and *xylB* genes driven by inducible promoter P*tet* | This work |
| pEZ-*RsXI*-*xylB* | pEZ containing codon-optimized *RsXI* and *xylB* genes driven by inducible promoter P*tet* | This work |
| pEZ-*RuXI*-*xylB* | pEZ containing codon-optimized *RuXI* and *xylB* genes driven by inducible promoter P*tet* | This work |
| **Strains** | | |
| ZM4 | *Z. mobilis* wild‑type strain | Lab stock |
| ZMP | ZM4 containing plasmid pZM41 | This work |
| ZMP-*EGFP* | ZMP containing plasmid pEZ-*EGFP* | This work |
| ZMP-*PiXI* | ZMP containing plasmid pEZ-*PiXI* | This work |
| ZMP-*RsXI* | ZMP containing plasmid pEZ-*RsXI* | This work |
| ZMP-*RuXI* | ZMP containing plasmid pEZ-*RuXI* | This work |
| ZMP-*PiXI*-*xylB* | ZMP containing plasmid pEZ-*PiXI*-*xylB* | This work |
| ZMP-*RsXI*-*xylB* | ZMP containing plasmid pEZ-*RsXI*-*xylB* | This work |
| ZMP-*RuXI*-*xylB* | ZMP containing plasmid pEZ-*RuXI*-*xylB* | This work |
| 8b | Recombinant ZM4 strain for xylose utilization, Tc^R^ Cm^R^ | [[3](#_ENREF_3)] |
| 8b-*PiXI*-*xylB* | 8b containing pEZ-*PiXI*-*xylB* | This work |
| 8b-*RsXI*-*xylB* | 8b containing pEZ-*RsXI*-*xylB* | This work |
| 8b-S1 | 8b-*RsXI*-*xylB* subcultured 1 time | This work |
| 8b-S8 | 8b-*RsXI*-*xylB* subcultured 8 times | This work |
| 8b-S11 | 8b-*RsXI*-*xylB* subcultured 11 times | This work |
| 8b-S15 | 8b-*RsXI*-*xylB* subcultured 15 times | This work |
| 8b-S19 | 8b-*RsXI*-*xylB* subcultured 19 times | This work |
| 8b-S38 | 8b-*RsXI*-*xylB* subcultured 38 times | This work |

1. Yang S, Mohagheghi A, Franden MA, Chou YC, Chen X, Dowe N, Himmel ME, Zhang M. Metabolic engineering of *Zymomonas mobilis* for 2,3-butanediol production from lignocellulosic biomass sugars. Biotechnol Biofuels. 2016;9(1):189.

2. Zhang M, Eddy C, Deanda K, Finkelstein M, Picataggio S. Metabolic engineering of a pentose metabolism pathway in ethanologenic *Zymomonas mobilis*. Science. 1995;267(5195):240-243.

3. Zhang M, Chou YC, Howe W, Eddy C, Evans K, Mohagheghi A. *Zymomonas* pentose-sugar fermenting strains and uses thereof. US Patent. 2007;US7223575
